# Transforming Speech-Language Pathology with AI: Opportunities, Challenges, and Ethical Guidelines

**DOI:** 10.3390/healthcare13192460

**Published:** 2025-09-28

**Authors:** Georgios P. Georgiou

**Affiliations:** 1 Department of Languages and Literature, University of Nicosia, Nicosia 2417, Cyprus; georgiou.georg@unic.ac.cy; 2 Phonetic Lab, University of Nicosia, Nicosia 2417, Cyprus

**Keywords:** artificial intelligence, speech-language pathology, clinical applications, ethical guidelines, health equity

## Abstract

Artificial intelligence (AI) is transforming the diagnosis, treatment, and management of speech-language disorders through advances in speech recognition, natural language processing, automated assessments, and personalized intervention. These tools have the potential to enhance clinical decision-making, improve diagnostic accuracy, and increase access to services for individuals with speech and language disorders, particularly in underserved populations. Despite this progress, adoption is challenged by data bias, lack of transparency, and limited integration into clinical workflows. To realize the potential of AI in communication sciences, both technical development and ethical safeguards are required. This paper outlines core applications, emerging opportunities, and major challenges in applying AI to speech-language pathology and proposes ethical principles for its responsible use.

## 1. Introduction

Artificial intelligence (AI) has transitioned in just two decades from a niche research interest to one of the most influential forces shaping healthcare. Within the field of communication sciences and disorders, this transformation has been especially pronounced [1]. Disorders of speech and language affect millions worldwide, creating significant barriers to education, employment, and social participation. Despite the recognized importance of early intervention, many individuals face prolonged delays in accessing care, often due to shortages of qualified speech-language pathologists (SLPs), geographic inequities, or the resource-intensive nature of traditional assessment and therapy [2,3].

Against this backdrop, AI tools are increasingly being explored as ways to aid in earlier detection, deliver more personalized interventions, and extend the reach of services to underserved populations [4]. Importantly, these advances align with broader healthcare goals of precision medicine, equitable access, and efficiency in clinical workflows [5].

The central aim of this paper is to provide a review of AI’s role in speech-language disorders. Specifically, it explores current applications, evaluates experimental studies of particular significance, outlines opportunities and challenges, and proposes concrete avenues for future research. In doing so, the paper not only synthesizes existing evidence but also sets a forward-looking agenda for responsible and impactful AI integration into communication sciences.

## 2. Applications of AI in Speech-Language Disorders

AI is redefining the landscape of communication disorder diagnostics and intervention across several critical areas.

### 2.1. Automated Assessment and Screening

Traditional assessment of speech and language disorders relies on standardized testing, observational analysis, and manual transcription of language samples. These approaches are effective but time-consuming and can be vulnerable to interrater variability. Recent work indicates that AI can augment these processes by extracting acoustic and linguistic patterns from short speech or text samples and by helping clinicians structure evidence from larger, multimodal records.

For developmental language disorder (DLD), Beccaluva et al. [6] demonstrate that machine learning (ML) models coupled with a speech data analysis tool can predict risk status from children’s language samples. Their work illustrates how engineered features and supervised models can support early identification workflows, and, more specifically, when embedded in tools designed for non-expert use, while also underscoring the importance of careful dataset curation and clinically meaningful evaluation. Georgiou & Theodorou [7] developed a neural network trained on Cypriot Greek children with DLD. Using perceptual and production measures and *k*-fold cross-validation, the model was evaluated with accuracy, precision, recall, F1, and ROC/AUC, achieving high performance across all metrics on unseen data. Variable-importance analysis showed that language production features contributed more than perception. The findings suggest ML can reliably distinguish DLD from typical development, supporting automatic assessment and screening.

For autism spectrum disorder (ASD), Ramesh & Assaf [8] tested classic ML models on speech transcripts from TalkBank (CHILDES/ASDBank) to distinguish children with ASD from typically developing peers, exploring data-driven screening using speech. The authors engineered 50+ linguistic features and tested five classifiers. Logistic Regression and Random Forest performed best, each reaching ~0.75 accuracy. Key predictors included mean length of utterance, mean length of turn ratio, part of speech patterns, and child word counts, with calls for larger, more diverse datasets. Their findings suggest speech-based ML can reliably aid timely ASD triage, with room to improve accuracy and generalizability alongside other modalities. 

Taken together, these studies indicate that AI can assist with screening and risk flagging across developmental conditions by processing short samples and surfacing patterns associated with impairment. Introductory guidance for clinicians on concepts and terminology further supports responsible interpretation and adoption [9], and broader work on early neurological detection highlights the translational potential of these methods when embedded within clinical pathways [5].

### 2.2. Speech Recognition and Transcription

Automatic speech recognition (ASR) has improved markedly with modern neural and transformer architectures, yet disordered speech remains challenging because training data are skewed toward typical speech patterns. Evidence shows that adapting models to conversational speech from individuals with speech-language disorders improves recognition performance [10]. More specifically, the study included speakers with diverse speech disorders, showing a consistent ASR accuracy gap between read and conversational speech for both a typical-speech ASR and participant-personalized models. Word error rates were higher for conversation, driven mainly by the linguistic complexity of utterances, with impairment severity also reducing accuracy (especially in unadapted models and for read speech in personalized models). Across nine examined factors (technical, linguistic, and impairment), linguistic attributes were most influential. Crucially, including conversational samples in training markedly improved recognition, showing the need for domain-specific, disorder-aware ASR training to handle conversational speech effectively. Complementary work targets dysarthric speech via training strategies and data augmentation. Wang et al. [11] showed that adversarial data augmentation during fine-tuning can enhance the robustness of pre-trained ASR to dysarthric variability, which improves recognition accuracy relative to naïve fine-tuning.

In clinical practice, better ASR has two direct benefits: (i) reduced transcription burden for documentation and research, and (ii) more scalable dataset creation for downstream analytics. Position papers and field studies with SLPs also note that automated transcription and summarization are among the most immediately useful capabilities for reducing administrative load when carefully integrated into workflows [12].

### 2.3. Voice and Acoustic Analysis

Objective acoustic analysis can complement perceptual ratings in the evaluation of voice disorders. Doctoral work on advanced speech analysis platforms describes the design and development of AI-driven pipelines that compute features such as jitter, shimmer, spectral tilt, and harmonic-to-noise ratios and use them within classification frameworks [13]. While specific accuracy figures vary by dataset and modeling approach, this line of research supports the feasibility of automated voice assessment and motivates clinical validation on diverse populations and recording conditions. In a case–control study using smartphone recordings, people who stutter (PWS) and age-/gender-matched controls (children, younger adults) produced a sustained vowel and two sentences [14]. Acoustic features were classified with a support vector machine (SVM), yielding high discrimination of PWS versus controls (accuracy: 88%) with comparable performance across speech tasks. Age-related differences in PWS were robust, as an SVM distinguished child from younger-adult PWS with 92% accuracy. Per-subject likelihood ratios derived via an auxiliary artificial neural network correlated significantly with clinical scales, supporting biological plausibility. 

Relatedly, in neurological voice analysis, Mittal & Sharma [15] tackled Parkinson’s Disease (PD) detection from speech by pairing data partitioning with principal component analysis (PCA) feature selection. The dataset was split into three equal parts, alternating emphasis on healthy vs. PD cases, and evaluated as a two-class problem using acoustic features. Three classifiers, namely logistic regression, weighted k-(nearest neighbors)NN, and Gaussian SVM, achieved baseline accuracies of 74.2%, 82.1%, and 85.0%. With PCA plus partitioning, performance improved to 80–89.23% (healthy-focused) and 85.2–90.3% (PD-focused), with SVM and k-NN strongest. The authors conclude the method is a viable approach for PD classification, highlighting the sensitivity of phonatory and prosodic markers to motor speech changes. Georgiou [5] situates such voice-based analytics within a broader biomedical engineering perspective on early detection and monitoring, and underlines their potential clinical value when embedded in validated pathways.

Overall, AI-based acoustic analysis can provide standardized, reproducible measures to support diagnosis and enable longitudinal monitoring (e.g., before/after interventions), though routine use requires attention to dataset representativeness, calibration, and interpretability.

### 2.4. Communication Aids and Augmentative Technologies

AI is also reshaping augmentative and alternative communication (AAC) and adjacent assistive technologies. Malviya & Rajput [16] reviewed and synthesized how AI can empower people with disabilities by improving communication efficiency and autonomy. They discussed capabilities such as predictive language modeling for faster message composition, context-aware vocabulary support, and more naturalistic speech synthesis, including voice personalization. While exact percentage gains depend on the system and user, the overall evidence base points to meaningful reductions in effort and improved user experience when predictive and personalization features are thoughtfully designed.

Bhardwaj et al. [17] reviewed how AI/ML, paired with AAC, are reshaping diagnosis and therapy for pediatric speech-sound and broader speech-language disorders. The authors argued that data-driven tools can enable earlier detection, personalize intervention, and augment clinicians’ decision-making, while AAC-enabled AI-powered apps can boost therapy engagement and parent involvement at home. Surveying current innovations and use cases, the paper positions AI as a complementary aid to clinical expertise rather than a replacement. Omoyemi [18] reviewed limitations of manual/predictive AAC interfaces and proposed a multimodal ML framework that fuses transformers/recurrent neural networks (RNN) for language and speech with a convolutional neural network (CNN)-based gesture recognition. Using simulated and pre-existing text, speech, and gesture datasets (no human participants), the system tokenizes/normalizes inputs and learns common AAC usage patterns to predict user intent and personalize suggestions over time. The reported outcomes included promising predictive accuracy and a roughly 30% reduction in message construction time, improving efficiency and user autonomy.

More broadly, studies of AI in remote clinical support and monitoring [19] and field reports from SLPs [20] echo that assistive AI must fit into real-world routines and uphold user control and transparency. Reviews on virtual therapy [21] similarly stress accessibility and engagement as central design goals for technology-supported communication interventions. Table 1 presents a summary of applications of AI in speech-language disorders.

## 3. Opportunities Created by AI in Speech-Language Disorders

**Figure 1:**
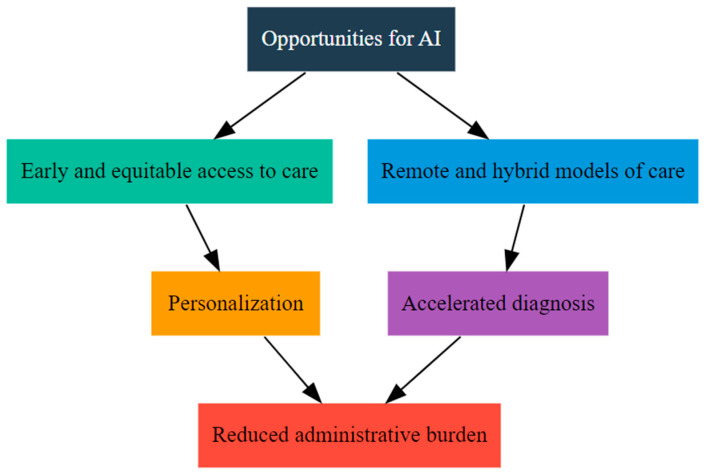
Opportunities created by AI in speech-language disorders.

AI introduces a suite of opportunities that address longstanding gaps in the field of communication disorders. Figure 1 provides a visual summary of the opportunities created by AI in speech-language disorders. It integrates the themes discussed in this section—early and equitable access, support for remote and hybrid care, data-driven personalization, research acceleration, and reduction in administrative burden—into a single schematic overview. It highlights how AI can address long-standing gaps in service provision and efficiency while aligning with broader healthcare goals of equity and precision medicine.

### 3.1. Early and Equitable Access to Services

AI-enabled screeners and mobile health tools can help triage large caseloads and shorten time-to-first-contact, especially in systems with long pediatric waits. Surveys show most SLP services report waiting lists, with waits averaging ~8 months and stretching to years in some settings. This indicates the need for scalable screening pathways in schools and primary care [22]. 

School-deployed, tablet-based screeners demonstrate that universal or near-universal classroom screening is feasible with solid sensitivity/specificity, offering a template for language-speech triage workflows. For example, Zugarramurdi et al. [23] introduced Lexiland, a gamified, tablet-based screener designed for universal, low-cost identification of early reading risk in school settings. The app replaces many spoken-response items with receptive, touch-only tasks embedded in a narrative, minimizing staff demands and making whole-class administration feasible. In a longitudinal sample of 616 kindergarteners, the authors used logistic regression with cross-validation to derive a reduced task set that predicts end-of-Grade-1 reading difficulties with 90% sensitivity and 76% specificity, and maintains 90% sensitivity with 61% specificity two years later. The study strengthens evidence that brief, device-based screeners can capture key predictors of reading outcomes at school entry while remaining scalable across iOS/Android.

### 3.2. Support for Remote and Hybrid Models of Care

Telepractice proved viable during and after the pandemic, reducing travel and scheduling barriers and helping maintain continuity when in-person care is scarce. Reviews document good acceptability and sustained use across pediatric and adult populations, with mixed but generally positive parent and clinician perceptions [24]. Targeted AI feedback can drive gains remotely. A single-case experimental study (ages 10–19) used an AI engine to predict clinician judgments and deliver automated /ɹ/ feedback; participants improved within sessions and generalized to untreated words, and families endorsed hybrid/at-home use, pointing to scalable intensity when clinicians are scarce [25].

Gale et al. [26] developed a tablet-administered sentence repetition screener that records a child’s spoken responses and uses ASR + ML to predict clinician scores. After task-specific ASR is adapted to child speech, the system computes item-level linguistic error features and concatenates them into a dimensional feature vector that summarizes performance across the test. A regression model trained on children (TD and neurodevelopmental groups, including DLD) predicted SLP gold-standard scores. Because administration and audio capture run on an iPad with automated scoring, the protocol is well-suited to remote or school-based prioritization, minimizing specialist time while keeping interpretable speech–language indicators (phonological accuracy, morphosyntax-sensitive errors, overall recall) visible for clinicians. Complementary studies demonstrate ASR-generated feedback can systematically shape speech clarity, and earlier feasibility work found an iPad app using ASR could deliver accurate and useful performance feedback; together supporting AI/ASR as the “automation layer” inside hybrid care [27].

Minority-language families face well-documented obstacles—language discordance, limited specialist availability, travel and scheduling burdens—so automation/ML-enabled remote screening can shorten time-to-identification and widen reach in underserved communities [28].

### 3.3. Data-Driven Personalization

Speech-language disorders are highly heterogeneous [29]. AI offers the ability to analyze multimodal data, namely, acoustic, linguistic, motor, and cognitive, and tailor therapy recommendations accordingly. This facilitates a shift from standardized protocols to dynamic and personalized treatment strategies aligned with individual needs and learning profiles.

Zhang et al. [30] designed a comprehensive induction procedure using audio-visual picture description, video retell, and conversation so that young children did not need to read. A two-stream deep model fused pronunciation (acoustic) with content (linguistic/semantic) indicators and reached 92.6% accuracy for DLD screening on ~2200 responses from more than 200 children. The indicator analysis highlighted which linguistic subsystems (e.g., fluency vs. expression efficiency) drove risk, a hook for tailoring the first therapy blocks to a child’s weakest domains. Toki et al. [31] used ML on a serious-game dataset capturing children’s speech-linguistic and cognitive signals. Clustering uncovered distinct performance profiles, and a logistic-regression model accurately distinguished neurodevelopmental risk. Those profiles and continuous risk scores can drive data-driven tailoring, e.g., selecting targets, cueing, and task difficulty/intensity per child, shifting screening from pass/fail to adaptive intervention planning.

From a data-science standpoint, methods for patient trajectory analysis can summarize heterogeneous longitudinal records and reveal response patterns that inform individualized planning [32]. This analytics layer helps clinicians reason over complex progress data and tailor interventions more precisely, especially when combined with domain guidance on model interpretation and deployment.

### 3.4. Research Acceleration

AI can extract meaningful patterns from large corpora of disordered speech, identify biomarkers of impairment, and reveal subgroup differences across diagnostic categories. These insights accelerate research into the mechanisms of speech-language disorders and inform novel approaches to classification and intervention. 

A bibliometric and visualization study tracked AI applications in Communication Sciences and Disorders from 1985 through December 2023 [33]. It found an average annual growth rate of 16.51%, with especially sharp acceleration from 2012 to 2023. The study cataloged 15,035 publications in Web of Science and Scopus, with 4375 meeting inclusion criteria. It showed a surge in AI-based work addressing autism, aphasia, dysarthria, PD, and dementia, using methods like ML, ASR, SVMs, and deep learning. This trend showcases how AI has expanded scientific exploration in the field.

As a result, the number of AI tools in the field has increased. Green et al. [34] outlined a wave of AI-driven clinical tools emerging rapidly: virtual therapists, interactive game-based platforms, chatbot partners, personalized therapy systems, intelligent assistants, voice cloning, ASR, eye-tracking applications, and brain–computer interfaces.

### 3.5. Reducing Administrative Burden

Administrative tasks, such as documentation, progress reporting, and data management, often consume a substantial portion of SLPs’ time, reducing the hours available for direct client care. Advances in AI now offer promising solutions to alleviate these burdens.

AI-driven tools can help SLPs reduce administrative workload by automating routine tasks [35,36]. For instance, speech-to-text systems can quickly convert spoken interactions into written form, streamlining session documentation and minimizing the need for manual note-taking. This not only improves efficiency but also frees clinicians to concentrate on client engagement during therapy. In addition, natural language processing and ML can be used to analyze therapy data and automatically generate progress summaries, highlighting key patterns, milestones, and areas of growth. Such capabilities make it easier for SLPs to produce clear, data-informed reports in less time. AI tools can also support task management by flagging notes or communications as urgent or routine according to set rules, and by automating repetitive paperwork and data entry, further easing the documentation burden.

## 4. Challenges to AI Adoption in Speech-Language Disorders

**Figure 2:**
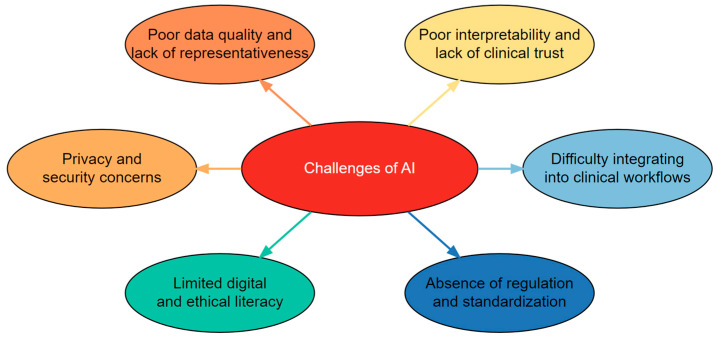
Challenges of AI adoption in speech-language disorders.

Despite its promise, several barriers complicate the adoption of AI in the communication sciences. Figure 2 summarizes the major challenges—data quality and representativeness, interpretability and clinical trust, workflow integration, regulatory uncertainty, limited digital literacy, and privacy/security risks—that complicate AI adoption in speech-language pathology. This schematic emphasizes that addressing these challenges requires coordinated efforts across technical development, clinical practice, and policy.

### 4.1. Poor Data Quality and Lack of Representativeness

AI models used in speech and language applications depend heavily on large, well-annotated datasets. However, such data often suffer from poor quality, limited annotation, and a lack of diversity, especially when compared across different populations. This issue is well-documented in reviews of clinical AI, which highlight how biased datasets can reinforce existing disparities rather than mitigate them [37].

A clear example in speech recognition: systems trained predominantly on standard accents or monolingual English outperform performance on dialect speakers and multilingual speakers, leading to worsened accuracy for underrepresented groups. A study of ASR systems found substantially higher error rates for Black speakers compared to others, underscoring how dataset bias negatively affects model fairness [38]. More broadly, analyses of clinical AI datasets show that over half originate from just the U.S. or China, often missing representation from other regions and demographic groups, resulting in models that struggle to generalize globally [39].

Consequently, these representational gaps reduce a model’s ability to generalize beyond its dominant training population, risking inequitable outcomes for speech-language services. For instance, tools trained on monolingual, middle-class English-speaking children may perform poorly when used with bilingual, dialect-speaking, or culturally diverse populations, thereby perpetuating rather than alleviating health disparities.

### 4.2. Poor Interpretability and Lack of Clinical Trust

Many clinicians remain hesitant about integrating AI systems in SLP when those systems operate as “black boxes”, which offer predictions or recommendations without clarity on how conclusions were reached. This is especially true in pediatric or neurological cases, where individual developmental history, environmental context, and atypical patterns require nuanced interpretation. Petch [40] documents clinicians’ lingering wariness in such contexts, which reinforces the need for transparency.

Explainable AI is positioned as a necessary corrective that offers interpretable outputs that practitioners can understand, validate, and relay to families or interdisciplinary teams. Abgrall et al. [41] report that improved transparency enhances clinician confidence and aids compliance with regulatory mandates demanding accountability in clinical decision-making.

However, the solution is not as simple as adding explanations. A systematic review by Rosenbacke et al. [42] found that while clear and concise explanations can increase clinicians’ trust, poorly constructed or overly complex ones may confuse clinicians or diminish their confidence in AI tools. Explanations must therefore be designed carefully, with clinical goals, user needs, and clarity at the forefront to be effective in real-world practice.

### 4.3. Difficulty Integrating into Clinical Workflows

AI tools developed within research environments often fall short of real-world clinical usability due to poor interface design, lack of integration with electronic health records (EHRs), and inflexible scoring processes, even when their technical performance is strong [43]. Research shows that poorly aligned interfaces disrupt clinician workflow, increasing cognitive load through excessive navigation and task switching across fragmented EHR screens. This friction not only undermines efficiency but also contributes to clinician burnout.

To be truly useful in practice, AI systems must be designed around the clinical workflow. Lessons from EHR deployment emphasize the need for user-centered design and seamless integration, so AI becomes a cognitive aid rather than a workflow obstacle [44]. Moreover, clinicians are far more likely to adopt AI features when they are embedded within familiar systems, such as EHRs supported by user-centered development, rapid feedback loops, and targeted training. One study of an AI-powered clinical search tool reported 93% adoption just three months after implementation, along with significantly higher user satisfaction and perceived time savings [45].

### 4.4. Absence of Domain-Specific Regulation and Standardization

Currently, there is a notable absence of dedicated regulatory or standardization pathways for AI tools in speech-language disorders, leaving critical questions unresolved, such as how to validate clinical efficacy, assign liability in cases of misdiagnosis, or manage ongoing model updates. Although U.S. and European regulators are beginning to develop frameworks for AI and software-as-a-medical-device, these mechanisms remain largely generalized and are frequently in draft form rather than fully implementable for specialized tools like those used in SLP.

For example, in the U.S., the FDA has issued guidance and an action plan for regulating AI/ML systems embedded in medical devices, but these remain broad and not yet fully operationalized for niche domains [46]. In Europe, the EU AI Act classifies medical AI tools as high-risk and imposes stricter governance, yet it functions alongside the established Medical Device Regulation (MDR) and does not provide communication-disorder-specific pathways [47]. Practical and technical challenges remain around continuous learning models, which can evolve after deployment, raising concerns about when re-approvals are required and how to monitor safety across different clinical environments [48].

This regulatory gray zone imposes uncertainty on both developers and clinicians and may hinder the adoption of AI in speech-language disorders practice due to concerns over safety, accountability, and integrity of patient care.

### 4.5. Limited Digital and Ethical Literacy

SLPs are rarely trained in AI, data ethics, or ML concepts [49]. Without adequate education, there is a risk of misusing AI tools or failing to recognize their limitations. Training programs must evolve to equip clinicians with the skills to critically evaluate and effectively use AI in practice.

A recent study highlighted that student SLPs often demonstrate overreliance on tools like ChatGPT, with limited critical appraisal skills or awareness of ethical pitfalls, pointing to significant gaps in education [50]. Without this foundational literacy, practitioners risk misusing AI or misinterpreting its outputs in ways that could compromise assessment validity, cultural responsiveness, or therapeutic integrity. Accordingly, it is imperative that graduate-level training and continuing education evolve to include formal instruction in AI concepts, algorithmic limitations, and ethical best practices, which will empower SLPs to thoughtfully evaluate and responsibly integrate AI tools into their professional workflows.

### 4.6. Privacy and Security Concerns

Voice and language data are inherently personal and often carry identifiable markers like accent, emotional tone, and speech idiosyncrasies. This makes them exceptionally sensitive and vulnerable to breaches when processed by AI systems. Berisha and Liss [51] emphasize the heightened privacy and security risks associated with health-related speech data, especially when leveraged by AI for analytics and documentation purposes. Moreover, recent investigations into voice-based cognitive assessments highlight how voice recordings can inadvertently disclose personal attributes, such as gender, ethnicity, or even emotional state, raising the likelihood of re-identification or misuse [52]. This amplification of risks demonstrates the critical need for strong data protection measures, transparent data governance frameworks, and secure storage protocols.

Given the heightened sensitivity of this domain, ensuring ethical standards also means involving participants, especially minors or neurodivergent individuals, in decisions about how their data is used and shared. Maintaining such participatory data governance helps preserve trust, empower clients, and align practices with evolving norms around consent, privacy, and autonomy.

## 5. Ethical Guidelines for AI in Speech-Language Disorders

### 5.1. Beneficence and Non-Maleficence

AI used for assessment, therapy support, or documentation in speech-language disorders should improve outcomes without introducing new harms. Practically, this means requiring (a) prospective, real-world validation in representative populations; (b) benchmarks against standard care; and (c) ongoing surveillance to detect data drift, subgroup performance gaps, and safety issues after deployment. Major health-AI guidance centers have safety, effectiveness, human oversight, and life-cycle governance as core duties [53]. Regulators likewise emphasize change control and post-market monitoring; for example, the U.S. FDA’s AI/ML SaMD Action Plan and subsequent guidance on Predetermined Change Control Plans (PCCPs) so models can be updated safely over time [54,55,56]. A concrete validation precedent comes from ophthalmology: a pivotal, prospective trial of an autonomous diagnostic system met pre-specified endpoints in primary care, illustrating the level of evidence appropriate for high-impact AI [57]. 

### 5.2. Transparency and Explainability

In pediatric and neurogenic communication-disorder contexts, clinicians and families need to understand what a system is doing and why. Ethical guidance calls for transparent documentation of training data, scope, limitations, and appropriate clinician-facing explanations to support informed consent and shared decision-making [53]. At the same time, scholarship cautions that popular post hoc “explanations” can mislead, where stakes are high and performance allows, inherently interpretable models are preferable to opaque models with superficial rationales [58,59]. For SLP-relevant tools (e.g., automated narrative scoring, dysarthria monitoring), explainability should translate into auditable features and clinician-checkable outputs that can be communicated in plain language to families.

### 5.3. Fairness and Equity

AI must not amplify disparities across dialects, languages, ages, or etiologies. Evidence from speech technology shows substantially higher ASR error rates for Black speakers across major systems [60], a caution for any SLP workflow that leans on transcription or voice analytics. Broader clinical AI also shows how proxy choices can entrench inequity; for example, a widely used risk algorithm that under-referred Black patients because it optimized for healthcare costs rather than needs [61]. Mitigations include representative training data, pre-deployment subgroup audits (e.g., bilingual/dialect-speaking children; acquired vs. developmental disorders), and participatory design with affected communities—all consistent with global guidance on equitable, trustworthy AI.

### 5.4. Accountability and Governance

Clear roles across the AI lifecycle are essential. Clinicians retain responsibility for care decisions; institutions must provide governance (procurement standards, risk assessment, incident response, training); and developers must document limitations, versioning, known failure modes, and update plans. U.S. regulators set expectations for real-world monitoring and change control [54,55,56]. Professional bodies offer operational principles and toolkits for AI governance in health systems [62]. In Europe, the EU AI Act (Regulation 2024/1689) classifies most medical AI as high-risk, layering requirements for risk management, data governance, quality management, human oversight, and transparency on top of MDR/IVDR on a phased implementation timeline [63]. 

### 5.5. Patient Autonomy and Informed Consent

Respect for autonomy requires disclosing when AI is used, explaining what it does, its limits, and how clinicians remain in the loop, and providing meaningful options to opt out where appropriate. Ethical frameworks in health AI place transparency and autonomy among core principles [53], and health-system guidance (e.g., NHS code of conduct for data-driven tech) operationalizes these duties for procurement and deployment [64]. Empirical studies indicate that patients generally want disclosure, and preferences about how much information is desired vary, indicating the need for consent materials tailored to patient needs [65,66].

### 5.6. Sustainability and Long-Term Impact

Ethical deployment weighs environmental and social effects. Training and serving large models can carry significant energy and carbon costs; rigorous studies show that model design, hardware, data-center efficiency, and grid mix can change the carbon footprint by orders of magnitude, and recommend reporting and reducing emissions [67,68]. Health systems have set net-zero trajectories; AI procurement should align to these plans [69]. Socially, leaders should monitor downstream effects on roles, clinician–patient relationships, and access, ensuring automation reduces documentation burden without eroding therapeutic contact or excluding lower-resource settings.

## 6. Future Avenues

Advancing ML for speech-language disorders now requires a decisive shift from proof-of-concept performance to practice-anchored evidence. Prospective, multi-site evaluations should compare AI-augmented screening and intervention workflows with current standards on outcomes that matter clinically (e.g., time-to-first-contact, functional gains, and subgroup false-negative rates), and they should follow established reporting guidance for AI interventions so results are reproducible and auditable. These frameworks, when applied rigorously, can help ensure that clinical trials specify intended use, human oversight, failure modes, and update plans; such elements have often been underreported in speech and voice applications [70].

Methodologically, the field needs representative, disorder-aware benchmarks that privilege spontaneous and conversational speech alongside controlled tasks, with explicit stratification by age, severity, dialect, bilingual status, and etiology. Fairness audits should be baked into model development and prospective evaluation, with subgroup metrics reported as primary outcomes rather than afterthoughts. Evidence that automatic transcription accuracy varies by dialect and speaker group highlights the risk of silently propagating inequities if data and metrics are not designed to surface them [71].

Because voice and language signals are uniquely identifying, privacy-preserving analytics must become first-class research targets. Work on speaker anonymization shows that it is possible to obfuscate identity while retaining linguistic content and intelligibility; adapting, stress-testing, and validating such approaches on clinical speech (including disordered phonation and atypical prosody) is a natural next step before large-scale deployment in telepractice and school-based screening [72].

Translation to routine care will depend on workflow-first design and robust lifecycle governance. Rather than standalone apps, models should be embedded in electronic records and school documentation systems, accompanied by monitoring plans that watch for data and concept drift, clear rollback criteria, and role-based accountability. A risk-management lens covering validity, safety, security, explainability, privacy enhancement, and bias mitigation offers a common language for SLPs, health-system leaders, and developers to align on deployment readiness and ongoing oversight [73].

Finally, sustainability should be treated as part of clinical effectiveness rather than an externality. Reporting the energy and compute “price tag” of training and serving models, prioritizing efficient architectures, and favoring on-device or distilled models where feasible will make AI-enabled screening and therapy more accessible in low-resource settings and less burdensome for health systems [74].

## 7. Conclusions

The landscape that emerges from this review is one of convergence: computational advances now meet long-standing clinical questions with a level of granularity previously out of reach. Across disparate lines of inquiry, a common thread is visible; signals extracted from everyday speech and language are increasingly mapped to clinically meaningful constructs, and documentation once confined to manual effort is becoming machine-readable. What emerges is not a replacement for expertise but a richer evidentiary layer around it, where patterns in acoustic, lexical, and interactional data coexist with professional judgment and lived experience.

The broader significance lies in repositioning communication sciences as a data-intensive domain without losing its human core. Evidence accumulates across screening, monitoring, documentation, and assistive communication, and the field’s discourse now routinely spans accuracy, validity, equity, accountability, privacy, and long-term impact. In this sense, AI functions as a lens, as it renders familiar clinical phenomena newly observable and comparable, drawing together methods, outcomes, and ethics within a shared analytic frame. This perspective offers a clearer view of what counts as progress: outcomes that are measurable, interpretable, and consonant with the dignity of people who rely on speech-language services.

## Figures and Tables

**Table 1:** Summary of applications of AI in speech-language disorders.

Application	Explanation
Automated assessment and screening	Software that quickly checks speech-language abilities and uses algorithms to flag possible difficulties or risk, so clinicians know who needs a fuller evaluation.
Speech recognition and transcription	Tech that turns spoken words into text in real time or from recordings; useful for documentation, captioning, and analyzing what was said.
Voice and acoustic analysis	Tools that measure properties of the voice and speech signal to detect or monitor disorders, fatigue, emotion, or treatment progress.
Communication aids and augmentative technologies	Devices and apps boards that help people with speech-language disorders produce messages and participate in conversation.

## Data Availability

No new data were created or analyzed in this study.
